# Structural and cognitive deficits in chronic carbon monoxide intoxication: a voxel-based morphometry study

**DOI:** 10.1186/1471-2377-13-129

**Published:** 2013-10-01

**Authors:** Hsiu-Ling Chen, Pei-Chin Chen, Cheng-Hsien Lu, Nai-Wen Hsu, Kun-Hsien Chou, Ching-Po Lin, Re-Wen Wu, Shau-Hsuan Li, Yu-Fan Cheng, Wei-Che Lin

**Affiliations:** 1 Departments of Diagnostic Radiology, Kaohsiung Chang Gung Memorial Hospital, Chang Gung University College of Medicine, 123 Ta-Pei Road, 83305, Niao-Sung, Kaohsiung, Taiwan; 2 Department of Biomedical Imaging and Radiological Sciences, National Yang-Ming University, Taipei, Taiwan; 3 Departments of Neurology, Kaohsiung Chang Gung Memorial Hospital, Chang Gung University College of Medicine, Kaohsiung, Taiwan; 4 Department of Radiology, Yuan's General Hospital, Kaohsiung, Taiwan; 5 Institute of Neuroscience, National Yang-Ming University, Taipei, Taiwan; 6 Departments of Orthopedic Surgery, Kaohsiung Chang Gung Memorial Hospital, Chang Gung University College of Medicine, Kaohsiung, Taiwan; 7 Departments of Internal Medicine, Kaohsiung Chang Gung Memorial Hospital, Chang Gung University College of Medicine, Kaohsiung, Taiwan

**Keywords:** Carbon monoxide intoxication, Cognitive deficits, Delayed encephalopathy, Magnetic resonance imaging, Voxel-based morphometry

## Abstract

**Background:**

Patients with carbon monoxide (CO) intoxication may develop ongoing neurological and psychiatric symptoms that ebb and flow, a condition often called delayed encephalopathy (DE). The association between morphologic changes in the brain and neuropsychological deficits in DE is poorly understood.

**Methods:**

Magnetic resonance imaging and neuropsychological tests were conducted on 11 CO patients with DE, 11 patients without DE, and 15 age-, sex-, and education-matched healthy subjects. Differences in gray matter volume (GMV) between the subgroups were assessed and further correlated with diminished cognitive functioning.

**Results:**

As a group, the patients had lower regional GMV compared to controls in the following regions: basal ganglia, left claustrum, right amygdala, left hippocampus, parietal lobes, and left frontal lobe. The reduced GMV in the bilateral basal ganglia, left post-central gyrus, and left hippocampus correlated with decreased perceptual organization and processing speed function. Those CO patients characterized by DE patients had a lower GMV in the left anterior cingulate and right amygdala, as well as lower levels of cognitive function, than the non-DE patients.

**Conclusions:**

Patients with CO intoxication in the chronic stage showed a worse cognitive and morphologic outcome, especially those with DE. This study provides additional evidence of gray matter structural abnormalities in the pathophysiology of DE in chronic CO intoxicated patients.

## Background

Acute carbon monoxide (CO) intoxication may induce diffuse white matter (WM) or gray matter (GM) damage, leading to chronic neuropsychiatric complications [1,2]. Patients recovering from acute CO intoxication may develop delayed encephalopathy (DE), which is characterized clinically by recurring neurologic or psychiatric symptoms punctuated by temporary asymptomatic periods (lucid intervals) of varying duration [3,4]. An increased duration of CO exposure, elevated carboxyhemoglobin (COHb), and damage to the globus pallidus or WM are risk factors for DE [5-7]. DE was associated with WM injury in one study [8], but more research is needed and the long-term prognosis of DE is uncertain. In particular, bilateral, symmetric, confluent areas of signal change in the periventricular WM and centrum semiovale is observed, as well as cytotoxic edema in studies emphasizing diffusion weighted imaging [9]. A diffusion tensor study revealed evidence of WM demyelination in DE and bridged its correlation with cognitive impairment [10].

Other research has emphasized cerebral perfusion in acute CO intoxication as appreciated with single photon emission computed tomography [11]. The alteration of cerebral perfusion in the development of DE is unknown. There is greater consensus that brain atrophy is found in chronic CO intoxication patients [12-14] as a result of either WM or GM damage. However, little is known about the association of regional GM atrophy on long-term cognitive outcomes, and the differences between DE and non-DE groups in this regard.

Voxel-based morphometry (VBM) is an assumption-free method that applies voxel-wise comparisons throughout the brain to detect differences in GM concentration in various study groups. Furthermore, it provides objective and operator-independent results. The use of VBM for brain-volume evaluation has been widely discussed [15,16], as it can help evaluate anatomic brain abnormalities in varying subgroups after CO intoxication and correlate the results with those of neuropsychological (NP) tests to assess disease involvement. This study evaluates differences among subgroups in long-term cognition, GM deficit, as well as the relationship between GM atrophy and cognitive decline after chronic CO intoxication.

## Methods

### Subjects

Twenty-two patients with CO intoxication (7 men and 15 women; mean age, 41.09 ± 9.25 years; range, 28–56 years) in the chronic phase (> 6 months) [17] and 15 age-, sex-, and education-matched healthy subjects (7 men and 8 women; mean age, 36.27 ± 10.41 years; range, 24–58 years) were recruited. Subjects received MRI studies and NP tests in the chronic stage in the CO intoxication group and at the time of enrollment in the control group. The mean duration between acute exposure and the acquisition time of MRIs for all patients was 25.05 ± 15.20 months.

Patients with a CO intoxication history were included in the study. A clear history of acute CO intoxication was defined as an episode of past exposure to burning charcoal or gas in an enclosed space and/or an elevated COHb level [18]. These patients either sought first aid at the emergency room of Chang Gung Memorial Hospital during acute CO intoxication and follow-up at the out-patient clinic (n = 17) or developed new symptoms after acute CO intoxication at the out-patient clinic (n = 5). The average initial COHb level of the patients (n = 17) was 15.95 ± 15.03% (range: 0.9-54.3%, mean of DE group: 22.8%, mean of non-DE group: 12.5%). All CO intoxicated patients awoke within 24 h and underwent hyperbaric oxygen therapy for several days. During the acute stage, 17 out of 22 patients underwent conventional MRI study. The exclusion criteria for this study included a history of neurologic or psychiatric illness, the presence of developmental disorders, the use of medication for unrelated conditions, and head injuries, which can affect the results of the neuropsychiatric or neuroimaging surveys [10].

The 22 CO intoxicated patients were further divided into two subgroups, based on the presence or absence of DE (11 in the non-DE group; 4 men and 7 women; mean age, 40.55 ± 9.45 years and 11 in the DE group; 3 men and 8 women; mean age, 41.64 ± 9.47 years), which was defined as a combination of events such as an initial change in consciousness due to CO exposure, recovery from the acute stage, lack of symptoms for periods of days to weeks, and exacerbation with neurologic and/or psychiatric symptoms [8].

The Chang Gung Memorial Hospital Ethics Committee approved the study and all patients and participants in the control group provided written informed consent.

### Neuropsychological (NP) tests

Patients and healthy subjects were administered subtests of the Wisconsin card sorting test (WCST) and the Wechsler Adult Intelligence Scale (WAIS).

#### Wisconsin card sorting test (WCST)

The WCST is commonly used to evaluate frontal executive function, such as concept formation, set shifting, and flexibility [19]. In this study, the WCST-128 card computerized version was administered by trained research assistants to decrease the complexity of the administered WCST and to increase the efficiency of data collection. The six WCST indices used for the analysis were perseverative response (PR), perseverative error (PE), non-perseverative error (NPE), percent conceptual level response, completed categories, and failure to maintain set [20].

#### Wechsler Adult Intelligence Scale (WAIS)

The WAIS, a family of tests created by David Wechsler to measure cognitive domains that contribute to intelligence (Wechsler, 1955, 1981, 1997), is used to assess a wide range of cognitive abilities and impairments. In this study, we used the full scale intelligence quotient measure from the Taiwanese version of the WAIS-III [21,22], which is based on the combined Verbal Comprehension Index (VCI), Perceptual Organization Index (POI), Working Memory Index (WMI), and Processing Speed Index (PSI) scores [23]. All of the participants finished picture completion and matrix reasoning, the subtests that comprised the POI, and the digit symbol and symbol search, which comprised the PSI.

### Magnetic Resonance Imaging (MRI) data acquisition

Magnetic resonance scanning was performed on a 3 T MRI system (Excite; GE Medical System) equipped with an 8-channel head coil. High resolution T1-weighted images were acquired parallel to the anterior commissure-posterior commissure line (AC-PC line), using 3-dimensional fluid-attenuated inversion-recovery fast spoiled gradient echo (3D FLAIR-FSPGR) sequences. The parameters were TR 9.492 ms, TE 3.888 ms, TI 450 ms, flip angle 20°, field of view (FOV) 24 × 24 cm, matrix size 512 × 512, 110 continuous slices with a slice thickness of 1.3 mm, and an in-plane spatial resolution of 0.47 × 0.47 mm.

### Imaging data processing

A T1 VBM approach based on Diffeomorphic Anatomical Registration Through Exponentiated Lie Algebra (DARTEL) was used for preprocessing and subsequent analyses of whole brain T1-weighted volumetric images [24,25]. Individual T1-weighted volumetric images were analyzed using the Gaser’s VBM8 toolbox (http://dbm.neuro.uni-jena.de/vbm/) with SPM8 (Statistical Parametric Mapping. Wellcome Department of Imaging Neuroscience, London, UK; available online at http://www.fil.ion.ucl.ac.uk/spm), implemented in Matlab 7.3 (MathWorks, MA, USA). DARTEL is a novel image registration method that uses large deformation in an inverse-consistent framework for spatial normalization in the SPM toolbox.

Briefly, all images were carefully checked by an experienced neuroradiologist to ensure that no scanner artifacts, motion problems, or gross anatomic abnormalities existed for each participant. The semiautomatic approach [26] was used to evaluate the quality of structural images, specifically for motion problems. Motion artifact was evaluated on individual tissue segment images and assigned a rating of none, mild, moderate, or severe. To reduce bias during VBM processing, images were excluded if their tissue segment images were given a rating of moderate or severe. The anterior commissure was set as the origin of imaging for each participant.

Whole brain native space T1-weighted images were normalized and the bias field corrected and segmented into GM, WM, and cerebro-spinal fluid (CSF) partitions, based on the same generative model [27]. Unified segmentation involved alternating between segmentation, bias field correction, and normalization to obtain local optimal solutions for each process. This procedure was further refined by applying an iterative hidden Markov field (HMRF) model [28] to improve the quality of tissue segmentation and minimize the influence of noise level.

To ensure accuracy of registration across subjects, the native space GM, WM, and CSF segments were imported into a rigidly aligned space and iteratively registered to group-specific templates generated from all images in this study through nonlinear warping using the DARTEL toolbox [25]. Recent studies have indicated that the DARTEL algorithm can improve intersubject registration and be useful for population-based research [29,30].

The deformation parameters obtained in the spatial normalization step were applied to individual tissue segments in a rigidly aligned space. The Jacobian determinants derived from nonlinear deformation for the correction of volume changes were also applied during the nonlinear spatial transformation to preserve the overall amount of each tissue segment after normalization. Since DARTEL worked on images with average brain size of total participants in this study, additional affine transformation between average group space and Montreal Neurological Institute ( MNI ) standard space was needed. Because the MNI standard space was constructed by affine registration of a number of subjects to a common standard coordinate system, it was reasonable to use only affine transformation to achieve a suitable alignment between these two spaces. The optimal-normalized tissue segments of each individual had an identical voxel size of 1.5 × 1.5 × 1.5 mm.

All normalized, segmented, and modulated MNI standard-space images were smoothed with an 8-mm Gaussian kernel prior to tissue volume calculation and voxel-wise group comparisons. Overall tissue volumes (i.e., GM, WM, and CSF) were estimated in mm^3^ by counting the voxels representing GM, WM, and CSF in standard space. The total intracranial volume (TIV) was determined as the sum of the three volumes.

### Statistical analysis

#### Analysis between groups

Statistical analysis was performed using the statistics computer software SPSS 12 (SPSS Inc, Chicago, IL). All data were given as the mean ± standard deviation (SD). The demographic and clinical characteristics of those in the patient and control groups were compared by analysis of variance (ANOVA) (for age and education). One-way analysis of covariance (ANCOVA) was used to compare TIV, GM volume, and WM volume, with age and sex as added covariates. Statistical differences in NP data, including the WCST and WAIS results between the two groups, were estimated by ANCOVA, with age, sex, and education as covariates. The threshold for statistical significance was *p* < 0.05.

Smoothed, modulated gray matter segments were analyzed with SPM8 within the framework of a General Linear Model (GLM). ANCOVA was performed with the covariation of age, sex, and TIV to investigate differences in regional GM volume between the two groups. All voxels with a GM probability value < 0.2 (range, 0–1) were eliminated to avoid possible partial volume effects around the margin between the GW and the WM. Nonstationary correction (part of the VBM toolbox) for correcting non-isotropic smoothness of the data was used to investigate group differences [31]. The differences in GM volume were compared between the following groups: 1) all patients vs. control group, 2) non-DE group vs. control group, 3) DE group vs. control group, and 4) non-DE group vs. DE group.

Because of the exploratory design of this study, strict criteria were used to obtain the findings. Low voxel-level thresholds (uncorrected *p* < 0.05) might sensitize the cluster inference for spatially extended and lower spatial resolutions. In contrast, high (uncorrected *p* < 0.001) contiguous voxel thresholds with a cluster size >50 might generate a higher spatial cluster resolution but result in the loss of spatial extent. In this study, the voxel-level threshold was set to an uncorrected *p* < 0.001 and a nonstationary cluster extent threshold of p < 0.05 corrected for multiple comparisons with family-wise error (FWE) [32] correction in to obtain precise findings with higher spatial cluster resolutions.

To minimize coordinate transformation discrepancies between the MNI and Talairach space, GingerALE provided by BrainMap (The BrainMap Development Team; available online at http://brainmap.org/ale/index.html) was used to transform MNI coordinates into Talairach coordinates. Anatomic structures of the coordinates representing significant clusters were identified based on the Talairach and Tournoux atlas [33].

#### Correlation analysis

Partial correlation analysis adjusted for age, sex, education, and TIV was performed to assess the correlation between NP test scores and areas with smaller volumes in all CO intoxicated patients compared to those in the control group. Regional GMV was extracted from the peak coordinate and correlated with NP test scores, with significance at *p* < 0.05 [34].

## Results

### Clinical characteristics and cognitive profiles among groups

The demographic characteristics of CO intoxicated patients and healthy controls are shown in Table 1. Seventeen of the 22 patients underwent conventional MRI during the acute stage with unremarkable findings (total: 9, DE: 3, non-DE: 6), globus pallidus signal change (DE: 7), and WM lesions (DE: 4). Three patients presented a coexistence of globus pallidus and white matter change. Symptoms of delayed encephalopathy (n = 11) included consciousness change (n = 3), Parkinsonism (n = 3), dystonia (n = 3), memory impairment (n = 1), and gait disturbance (n = 1).

**Table 1:** Demographic characteristics of COI patients and normal controls

	**Patients with chronic COI**			**Normal group**	**F**^***†***^	***p***^***† ***^**value**
	**Non-DE group**	**DE group**	**All patients**			
Definition	without DE	with DE	Chronic COI	Healthy control		
Number of cases	11	11	22	15		
Sex (n = men/women)	4/7	3/8	7/15	7/8		
Age (years)	40.55 ± 9.45	41.64 ± 9.47	41.09 ± 9.25	36.27 ± 10.41	2.193	0.148
Education	13.00 ± 1.70 ^#^	12.25 ± 2.44 ^§^	12.67 ± 2.03	15.20 ± 1.47 ^#§^	16.213	**0.000**
Duration of follow-up (month)	27.73 ± 13.66	22.36 ± 16.82	25.05 ± 15.20	-		
Conventional MRI findings during the acute stage (n = normal/basal ganglia/WM lesions)	6/0/0	3/7/4	9/7/4	-		
COHb% during the acute stage	12.50 ± 13.02	22.8 ± 17.60	15.95 ± 15.03	-		
**Total intracranial volume (TIV) (cm**^**3**^**)**	1459.26 ± 136.37	1469.58 ± 148.12	1464.42 ± 139.04	1473.29 ± 157.60	0.135	0.715
Gray matter (GM)	670.41 ± 56.26	662.79 ± 69.09	666.60 ± 61.61	683.34 ± 65.58	0.034	0.856
White matter (WM)	490.27 ± 54.82	498.18 ± 54.61	494.22 ± 53.55	495.54 ± 53.09	0.086	0.771
**WCST**						
Perseverative response (PR)	17.60 ± 17.20	17.00 ± 14.92	17.33 ± 15.76	6.93 ± 3.56	3.705	0.064
Perseverative error (PE)	14.80 ± 12.43	14.25 ± 11.76	14.56 ±11.78	6.73 ± 3.10	3.943	0.057
Non-perseverative error (NPE)	9.30 ± 3.62	10.00 ± 2.73	9.61 ±3.18	7.27 ± 3.71	2.923	0.098
Percent conceptual level response	49.85 ± 23.07 ^#^	52.35 ± 22.75	50.96 ±22.29	71.98 ± 13.29 ^#^	7.245	0.012^*^
Completed categories	2.40 ± 1.51	2.13 ± 1.36	2.28 ±1.41	3.73 ± 1.33	4.069	0.053
Failure to maintain set	0.40 ± 0.70	0.63 ± 0.74	0.50 ± 0.71	0.47 ± 0.74	2.023	0.166
**WAIS**						
Picture completion	13.80 ± 3.77	13.13 ± 5.84	13.50 ±4.66	17.93 ±3.43	4.766	0.038^*^
Digit symbol	65.60 ± 20.10 ^#^	60.50 ± 16.01 ^§^	63.33 ±18.06	92.20 ± 14.90 ^#§^	10.475	0.003^**^
Symbol search	28.30 ± 6.95 ^#^	22.88 ± 14.20 ^§^	25.89 ±10.78	40.00 ± 8.13 ^#§^	6.765	0.015^*^
Matrix reasoning	14.00 ± 4.62	12.13 ± 4.26	13.17 ±4.44	17.40 ± 6.13	0.339	0.565

Age and education data were compared by ANOVA. TIV, GM, and WM data were compared by ANCOVA after controlling for age and sex.

NP data (WAIS and WCST) were compared by ANCOVA after controlling for age, sex, and education.

Data are presented as mean ± SD.

F^*†*^ and p^*†*^ represent the comparison between all COI patients vs. the normal control group.

^#^Significant differences between the normal group and the non-DE group in the post-hoc analysis with *Bonferroni’s* correction.

^§^Significant differences between the normal group and the DE-group in the post-hoc analysis with *Bonferroni’s* correction.

Boldfaced values represent significant differences (*p* ≤ 0.05).

*Abbreviations*: *WAIS* Wechsler Adult Intelligence Scale; *WCST* Wisconsin Card Sorting Test.

^*^p < 0.05; ^**^p < 0.001.

The mean interval between initial CO exposure and the date of the MRI study and NP testing was 27.73 ± 13.66 months (range, 6–45 months) in the non-DE group and 22.36 ± 16.82 months (range, 6–51 months) in the DE group. The TIV was 1459.26 ± 136.37 cm3, 1469.58 ± 148.12 cm3, and 1464.42 ± 139.04 cm3 in the non-DE, DE, and control groups, respectively.

Except for lower education level (F = 16.213; *p* < 0.05), there were no significant differences in age, sex, and regional brain volume between patients with and those without DE. All patients, regardless of DE, performed significantly worse than those in the control group in the WCST percent conceptual level response, and in the picture completion, digit symbol, and symbol search tests of the WAIS (*p* < 0.05) (Table 1). Compared individually in the post-hoc analysis to those in the control group, those in the non-DE group showed a lower performance level in percent conceptual level response, digit symbol, and symbol search tests, whereas patients in the DE group showed a lower performance level in only the digit symbol and symbol search tests.

### Regional gray matter volume (GMV) aberration among groups

The location and extent of regions with significant differences in GMV are presented in Table 2.

**Table 2:** Regions of statistically significant lower GMV in chronic COI patients compared to those of controls

**Gray matter volume**	**Anatomical regions**	**x**	**y**	**z**	**Brodmann’s area**	**Cluster size**	**T value**
Normal > All patients	L Lateral Globus Pallidus	−12	8	2	-	616	5.22
	L Claustrum	−24	21	2	-	813	5.18
	R Amygdala	26	−9	−15	-	676	4.97
	R Caudate Body	9	14	8	-	374	4.78
	L Hippocampus	−33	−14	−15	-	291	4.75
	R Putamen	27	20	5	-	540	4.47
	R Supra-marginal Gyrus	50	−42	41	40	198	4.31
	L Superior Frontal Gyrus	−21	54	24	9	218	4.31
	L Post-central Gyrus	−41	−23	42	2	290	4.2
	L Hypothalamus	0	−6	−11	-	283	4.18
Normal > Chronic	L Post-central Gyrus	−42	−23	41	2	51	3.86
Normal > Delay	L Claustrum*	−24	21	2	-	2116	5.88
	R Amygdala*	29	−9	−17	-	1177	5.81
	R Caudate Body	9	14	6	-	1192	5.14
	L Hippocampus	−33	−12	−15	-	488	4.8
	R Inferior Parietal Lobule	51	−44	41	40	233	4.35
	L Superior Frontal Gyrus	−23	54	26	9	194	3.98
	L Medial Frontal Gyrus	−2	56	9	9	120	3.9
	R Anterior Cingulate	8	48	−8	32	119	3.66
	L Post-central Gyrus	−38	−21	53	3	80	3.62
	L Mammillary Body	2	−8	−11	-	63	3.59
Chronic > Delay	L Anterior Cingulate	−2	48	−11	32	2301	5.06
	R Amygdala	29	−11	−18	-	112	3.73

Voxel-based morphometry of COI patients compared to those of normal controls at an uncorrected *p* value (<0.001).

* Indicated for statistical threshold: uncorrected p < 0.001 with a cluster extent correction FWE-corrected p value < 0.05.

(x, y, and z) refer to the Montreal Neurological Institute coordinates. The T-value is determined by dividing the estimated regression coefficient by its standard error.

*Abbreviations*: *R* Right; *L* Left.

#### Comparison between all patients and controls

**Figure 1:**
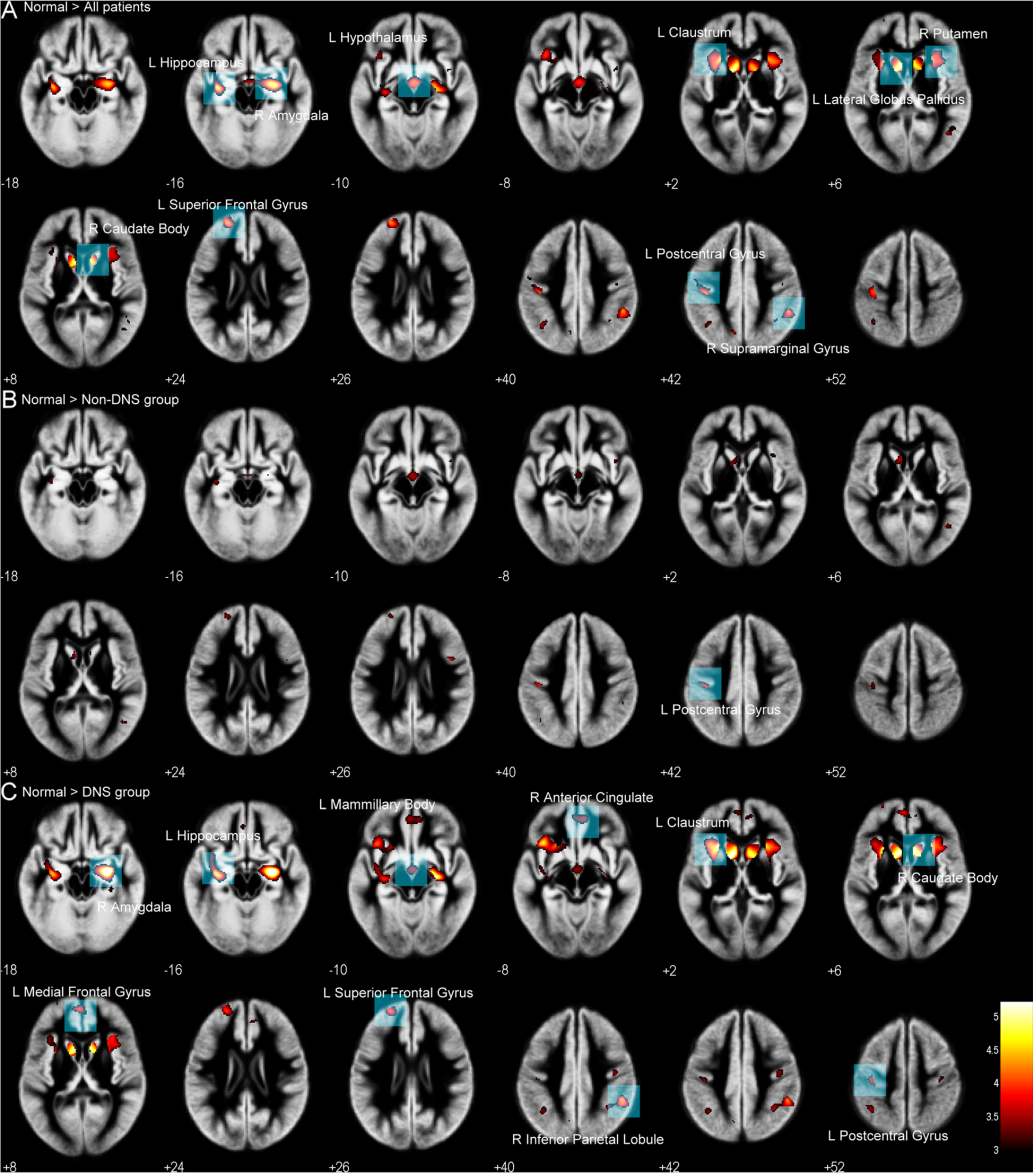
**Lower gray matter volumes (GMVs) in chronic CO-intoxicated patients vs normal subjects, with highlighted significant areas. (A)** Compared to healthy controls, all chronic CO-intoxicated patients showed significantly lower GMV in the bilateral basal ganglia, left claustrum, right amygdala, left hippocampus, bilateral parietal, and left frontal lobes. **(B)** Only one significant difference in GMV was observed in the left post-central gyrus between the healthy control and the non-DE groups. **(C)** The DE group showed significantly lower GMV in the left hippocampus, left mammillary body, left claustrum, right amygdala, right anterior cingulate, right caudate body, and left frontal and bilateral parietal lobes. Among these areas, the clusters of left claustrum and right amygdala survived from statistical threshold of FWE corrected p < 0.05.

During the chronic stage, all patients showed lower NP subtest results and significantly lower GMV in the left lateral globus pallidus, left claustrum, right amygdala, right caudate body, left hippocampus, right putamen, left hypothalamus, and left frontal and bilateral parietal lobes (uncorrected *p* < 0.001) compared to those in the control group (Figure 1A).

#### Comparison between the DE and control groups and between the non-DE and control groups

Based on a nonstationary cluster extent threshold of p < 0.05 corrected for multiple comparisons with FWE, there was a significantly lower GMV in the left claustum and right amygdala of those in the DE group, compared to those in the control group. By using a p value of 0.001 (uncorrected), patients with DE were shown to have a lower GMV in the right caudate body, left hippocampus, right inferior parietal lobule, left superior frontal gyrus, left medial frontal gyrus, right anterior cingulate, left post-central gyrus, and left mammillary body (Figure 1C). The non-DE group had a lower GMV in the left post-central gyrus compared to those in the control group (uncorrected p < 0.001) (Figure 1B).

#### Comparison between DE and non-DE groups

**Figure 2:**
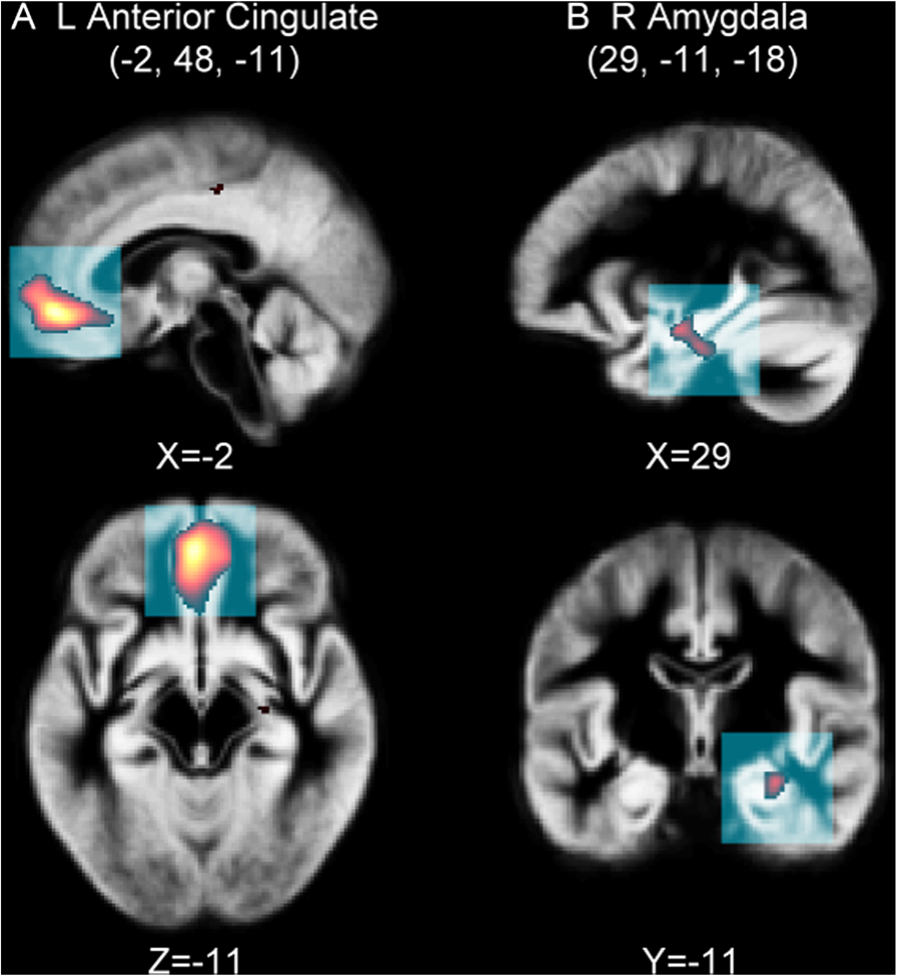
**Gray matter volume difference between DE and non-DE group.** The DE group showed significantly lower GMV in the **(A)** left anterior cingulate and **(B)** right amygdala, compared to non-DE group, using an uncorrected *p* < 0.001 and cluster size >50 voxels.

Compared to patients in the non-DE group, those in the DE group had a lower GMV in the left anterior cingulate and right amygdala (Figure 2).

### Relationship between cognition function and gray matter volume

The relationship between the three WAIS scores (Picture Completion, Digit symbol, and Symbol search) and the significantly lower GMV in the all patient groups compared to the control group showed significantly positive correlations (*p* < 0.05) (Table 3). The poor picture completion score correlated with a low GMV in the left lateral globus pallidus, left post-central gyrus, and left hypothalamus. The low digit symbol score correlated with a low GMV in the left hypothalamus, whereas a low symbol search score correlated with a low GMV in the right putamen.

**Table 3:** Correlation between NP variables and reduction in gray matter volume for all subjects

		**NP variables**	
**Anatomic region**	**Picture completion**	**Digit symbol**	**Symbol search**
**L Lateral Globus Pallidus**	0.4209*	0.2890	0.3464
L Claustrum	−0.0258	0.2587	0.3634
R Amygdala	0.0862	0.1735	0.1602
R Caudate Body	0.3363	0.2745	0.3060
L Hippocampus	−0.1296	0.1511	0.1698
**R Putamen**	0.0414	0.1965	0.4277*
R Supra-marginal Gyrus	−0.0142	0.1528	0.0144
L Superior Frontal Gyrus	−0.0814	−0.0252	0.1049
**L Post-central Gyrus**	0.3695*	0.2292	0.1735
**L Hypothalamus**	0.3702*	0.4083*	0.3328

The correlation is demonstrated as r-value.

*p < 0.05.

All data shown were obtained by partial correlation after controlling for age, sex, educational level, and total intracranial volume.

*Abbreviations*: *R* Right; *L* Left.

Furthermore, no significant correlation was found between the NP results and the low GMV detected by subgroup analysis in the DE, non-DE, and control groups.

## Discussion

To date, the relationship between structural changes and altered cognitive function in DE and non-DE patients has not been fully studied. In this first VBM study which compares these two groups, morphological differences were found between DE and non-DE patients, and many of the structural deficits of DE patients correlated with their lower level of cognition. The DE group demonstrated lower scores in the WAIS tests, with poor perceptual organization (Picture Completion) [35], and slow processing speed (Digit Symbol and Symbol Search) [35]. Carbon monoxide poisoning-related cognitive impairment includes impaired memory and executive function [36], reduced mental processing speed, and decreased intellectual function [14], which are consistent with the NP results in this study. However, the memory function has not been assessed in this study that may be seen as a limitation. The results of this study further support previous findings indicating that delayed CO encephalopathy might impair the frontal executive function and persist even after the recovery of other neurological deficits [37]. Increased structural alterations in DE patients corroborate the NP sequelae in patients with chronic status after CO intoxication. Long-term follow-up for this particular group is required.

Consistent with the hypothesis posed in this study, structural differences between the DE and non-DE groups were found. In the non-DE group, only one significant cluster of lower GMV than controls was observed, it’s location in the left post-central gyrus. In contrast, structural abnormalities in the DE group included lower volume of the left claustrum, right amygdala, right caudate body, left hippocampus, right inferior parietal lobule, left superior frontal gyrus, left medial frontal gyrus, right anterior cingulate, left post-central gyrus, and left mammillary body. Previous studies report generalized brain atrophy occurring in chronic CO intoxicated patients, with volume reductions in the fornix [12], hippocampus [14], corpus callosum [13], basal ganglia, and cerebral and cerebellar cortex. However, detailed lobar location of cortical involvement has never been studied [38]. In this study, the impact of DE on the brain’s gray matter, which had not been documented before, was clearly demonstrated. This VBM study provides a highly objective method for clarifying significant brain atrophy in the DE group, compared to the non-DE counterpart.

The pathogenesis of DE after CO intoxication is likely multifactorial, involving mitochondrial damage combined with hypoxemia and hypoperfusion during the acute stage [39-41], as well as glutamatergic excitation [42] and lipid peroxidation after return to CO-free air [43]. Although the exact mechanism in the development of DE is unclear, a COHb concentration level that is higher in the DE group than in the non-DE group in this study might increase the occurrence of delayed symptoms and suggest a more obvious effect on the brain [5-7]. The pathologic hallmark is extensive demyelination, and current theories for the pathogenic mechanism include direct toxicity effects of CO, cerebral blood vessel damage, cerebral edema, and a hypersensitive reaction [44]. Individual self-protective factors like tolerance to hypoxia and hypoperfusion or resistance to CO cytotoxic action in WM [4,8,10], may be responsible for the presence and variable duration of clear periods in DE.

In this study, two locations display a lower GMV in the DE group compared to the non-DE group: the left anterior cingulate (BA32) and the right amygdala. Both are a part of the brain limbic system. Past neuroimaging research suggests the anterior cingulate cortex to be part of the circuit involved in a type of attention [45] that regulates both cognitive and emotional processing [46]. The affective subdivision of the anterior cingulate connects to the amygdala [47], and modulation of the amygdala-anterior cingulate connections seems to be a key substrate of emotional attention bias [48,49]. Lesions involving the anterior cingulate and amygdala is believed to disrupt and cause affective biases [50]. In this study, the DE group presented lower levels of performance in digit symbol and symbol search, indicating a slower processing speed, which is thought to be connected with a poor attention function [51]. This result is consistent with previous concept, even though there is no significant correlation between the cluster volume in the left anterior cingulate, right amygdala, and the reported NP tests.

Although this study offers valuable insights into the cortical involvement in CO intoxication issues, it nevertheless has some limitations. First, our sample size is too small for definite conclusion of DE and non-DE. Patients without DE were difficult to contact and often rejected invitations to join the non-invasive research because they have little concern about following-up results in the chronic stage. Therefore, the number of patients in the non-DE group was smaller than the number of patients in the DE group, even though the incidence of DE is approximately 10% in all CO-intoxicated patients [52]. Second, varying treatments for CO-intoxicated patients exist, such as whether they receive hyperbaric oxygen therapy or not. Moreover, the initial laboratory data were not thoroughly collected for every subject in the emergency room. Therefore, initial disease severity could not be compared to long-term outcomes to define a clearer relationship.

## Conclusion

In conclusion, cognitive impairment and morphologic deficits were found to be present in patients with CO intoxication even after long-term follow-up. Patients in the DE group accomplished with more imaging abnormalities and higher COHb level will express worse neuropsychiatric outcome. In addition to WM injury, the GM microstructure damage also has clinical implication and additional psychiatric research in CO intoxication should prove quite beneficial.

## Abbreviations

CO: Carbon monoxide; DE: Delayed encephalopathy; VBM: Voxel-based morphometry; GM: Gray matter; GMV: Gray matter volume; WM: White matter; NP: Neuropsychological; WCST: Wisconsin card sorting test; WAIS: Wechsler Adult Intelligence Scale; TIV: Total intracranial volume; BA: Brodmann’s areas.

## Competing interests

Financial competing interests

• In the past five years have you received reimbursements, fees, funding, or salary from an organization that may in any way gain or lose financially from the publication of this manuscript, either now or in the future? Is such an organization financing this manuscript (including the article-processing charge)? **No.**

• Do you hold any stocks or shares in an organization that may in any way gain or lose financially from the publication of this manuscript, either now or in the future? **No.**

• Do you hold or are you currently applying for any patents relating to the content of the manuscript? Have you received reimbursements, fees, funding, or salary from an organization that holds or has applied for patents relating to the content of the manuscript? **No.**

• Do you have any other financial competing interests? **No.**

Non-financial competing interests

• Are there any non-financial competing interests (political, personal, religious, ideological, academic, intellectual, commercial or any other) to declare in relation to this manuscript? **No.**

## Authors’ contributions

Study concepts: HLC. Study design: HLC and WCL. Data acquisition: HLC, PCC, NWH, RWW, SHL, and YFC. Data analysis and interpretation: HLC, PCC, WLC, NWH, and KHC. Statistical analysis: HLC, PCC, KHC, and CPL. Manuscript preparation: HLC, and PCC. Manuscript editing: HLC, and WCL. Manuscript review: CHL, and WCL. All authors read and approved the final manuscript.

## Pre-publication history

The pre-publication history for this paper can be accessed here:

http://www.biomedcentral.com/1471-2377/13/129/prepub
